# Mucolipin Co-deficiency Causes Accelerated Endolysosomal Vacuolation of Enterocytes and Failure-to-Thrive from Birth to Weaning

**DOI:** 10.1371/journal.pgen.1004833

**Published:** 2014-12-18

**Authors:** Natalie N. Remis, Teerawat Wiwatpanit, Andrew J. Castiglioni, Emma N. Flores, Jorge A. Cantú, Jaime García-Añoveros

**Affiliations:** 1 Driskill Graduate Program in the Life Sciences (DGP), Northwestern University Feinberg School of Medicine, Chicago, Illinois, United States of America; 2 Department of Anesthesiology, Northwestern University Feinberg School of Medicine, Chicago, Illinois, United States of America; 3 Northwestern University Interdepartmental Neuroscience (NUIN) graduate program, Chicago, Illinois, United States of America; 4 Departments of Neurology and Physiology, and Hugh Knowles Center for Clinical and Basic Science in Hearing and Its Disorders, Northwestern University Feinberg School of Medicine, Chicago, Illinois, United States of America; University of Michigan, United States of America

## Abstract

During the suckling period, intestinal enterocytes are richly endowed with endosomes and lysosomes, which they presumably utilize for the uptake and intracellular digestion of milk proteins. By weaning, mature intestinal enterocytes replace those rich in lysosomes. We found that mouse enterocytes before weaning express high levels of two endolysosomal cation channels, mucolipins 3 and 1 -products of *Trpml3* and *Trpml1* genes; moreover neonatal enterocytes of mice lacking both mucolipins (*Trpml3^−/−^*;*Trpml1^−/−^*) vacuolated pathologically within hours of birth and remained so until weaning. Ultrastructurally and chemically these fast-forming vacuoles resembled those that systemically appear in epithelial cells of mucolipidosis type IV (MLIV) patients, which bear mutations in *Trpml1*. Hence, lack of both mucolipins 1 and 3 causes an accelerated MLIV-type of vacuolation in enterocytes. The vacuoles were aberrant hybrid organelles with both endosomal and lysosomal components, and were not generated by alterations in endocytosis or exocytosis, but likely by an imbalance between fusion of lysosomes and endosomes and their subsequent scission. However, upon extensive vacuolation enterocytes displayed reduced endocytosis from the intestinal lumen, a defect expected to compromise nutrient uptake. Mice lacking both mucolipins suffered a growth delay that began after birth and continued through the suckling period but recovered after weaning, coinciding with the developmental period of enterocyte vacuolation. Our results demonstrate genetic redundancy between lysosomal mucolipins 3 and 1 in neonatal enterocytes. Furthermore, our *Trpml3^−/−^*;*Trpml1^−/−^* mice represent a polygenic animal model of the poorly-understood, and often intractable, neonatal failure-to-thrive with intestinal pathology. Our results implicate lysosomes in neonatal intestinal pathologies, a major cause of infant mortality worldwide, and suggest transient intestinal dysfunction might affect newborns with lysosomal storage disorders. Finally, we conclude that mucolipin-endowed lysosomes in the young play an evolutionarily-conserved role in the intracellular digestion of maternally-provided nutrients, whether milk in mammals or yolk in oviparous species.

## Author Summary

Intestinal digestion is very different before and after weaning. In adults, extracellular enzymes in the lumen of digestive tract digest proteins and the enterocytes lining the intestine absorb the resulting amino acids. During suckling, proteins reach the intestinal lumen intact, are taken (endocytosed) by enterocytes and degraded inside them. For this intracellular digestion enterocytes prior to weaning have specialized lysosomes with digestive enzymes. Lysosomes are also of biomedical relevance because their partial dysfunction causes ∼50 genetic disorders with a range of symptoms (Lysosomal Storage Disorders; LSDs). We found that enterocytes prior to weaning express two related proteins implicated in certain LSDs (mucolipins 1 and 3) and that their co-absence causes pathological vacuolation of enterocytes, diminished apical endocytosis from the intestinal lumen, diarrhea and delayed growth (failure to thrive) from birth to weaning. Our results implicate lysosomes in neonatal intestinal disorders, a major cause of infant mortality, and suggest transient intestinal dysfunction might affect newborns with LSDs. Hence, we link two large sets of disorders that are presently considered and treated as unrelated. Finally, we propose that the special mechanisms for the uptake and digestion of maternal milk are not unique to mammals, as embryos of oviparous species use a similar mechanism for the digestion of maternally-provided yolk.

## Introduction

In mammals, including humans, digestion fundamentally differs between the suckling and post-weaning periods [1], [2]. In adults, extracellular proteases digest ingested proteins in the lumen of the stomach and intestine and intestinal enterocytes uptake the resulting amino acids. However, during suckling the stomach has a high pH and lacks pepsin, and as a consequence proteins from ingested milk pass intact to the intestines, where they are endocytosed by enterocytes for intracellular digestion in lysosomes [1]–[4]. For this unique form of feeding, perinatal enterocytes generate *de novo* a specialized system of endosomes and lysosomes that lasts until weaning, when they are replaced by adult enterocytes [5], [6].

Mucolipins are cation channels present in the membranes of lysosomes and late endosomes [7]–[9]. Mammals have three mucolipin paralogs, encoded by the genes *Trpml1*, *2* and *3*. Mutations in human *Trpml1* (also known as *Mcoln1*) cause mucolipidosis type IV, a lysosomal storage disorder characterized by severe psychomotor retardation and ophthalmological abnormalities that typically appear months after birth but within the first year of life [10], [11]. Mice lacking mucolipin 1 (*Trpml1* ^−/−^) develop similar symptoms also about six months after birth (that is, with a similar onset in absolute time but at a much later developmental stage with respect to humans) [12], [13]. Cells of MLIV patients and *Trpml1* ^−/−^ mice display enlarged lysosomal vacuoles that are largely empty or accumulate various undigested substances, depending on cell type, but that typically contain membranous bodies with concentric lipid membranes [11], [14]. The slow onset of these subcellular abnormalities pose an obstacle to elucidating how the pathological vacuolation occurs in the absence of mucolipin 1, and have also led to the suspicion that other channels, perhaps mucolipins 2 or 3, may partially compensate for the loss of mucolipin 1.

Unlike the ubiquitously expressed mucolipin 1, the paralog mucolipin 3 is expressed in a restricted set of cell types which include hair cells of the inner ear and melanocytes of the skin [15]–[17]. Dominant point mutations in the mouse *Trpml3* gene result in hyperactive ion channels that are lethal to cells expressing them, causing deafness due to loss of hair cells and hypopigmentation presumably due to loss of melanocytes in the *varitint-waddler Va* and *Va^J^* mice [16]–[20]. These gain-of-function mutations, however, do not clarify the role mucolipin 3 may play in the restricted set of cells expressing it.

The relevance of mucolipins extends beyond the *varitint-waddler* mice and MLIV to many other diseases caused by mutations in other genes (such as sphingomyelinases for Nieman-Pick); the pathologically-accumulated lipids inhibit mucolipin 1 channels, which disrupts lysosomal trafficking and thus aggravates the cellular pathology of these diseases [21], [22]. Hence, it is pressing to elucidate the role of mucolipins in lysosomes and the nature of the lysosomal abnormalities caused by their dysfunction.

Here we find that the lysosome-containing enterocytes of the suckling period express mucolipin 3 and upregulate mucolipin 1, and that mice lacking both mucolipins (but not only one of the two) suffer delayed growth (faltering) together with pathological vacuolation of enterocytes throughout the period of suckling, until weaning. The vacuolated enterocytes assemble within hours a pathological organelle with both endosomal and lysosomal components that is similar to the pathological vacuoles that form in epithelial cells of MLIV patients within months. Following enterocyte vacuolation is a reduction of endocytosis from the intestinal lumen, a presumed cause for a deficiency in nutrient uptake that would account for the delayed growth.

## Results

### Enterocytes of neonatal (suckling) but not adult small intestines express *Trpml3*

**Figure 1:**
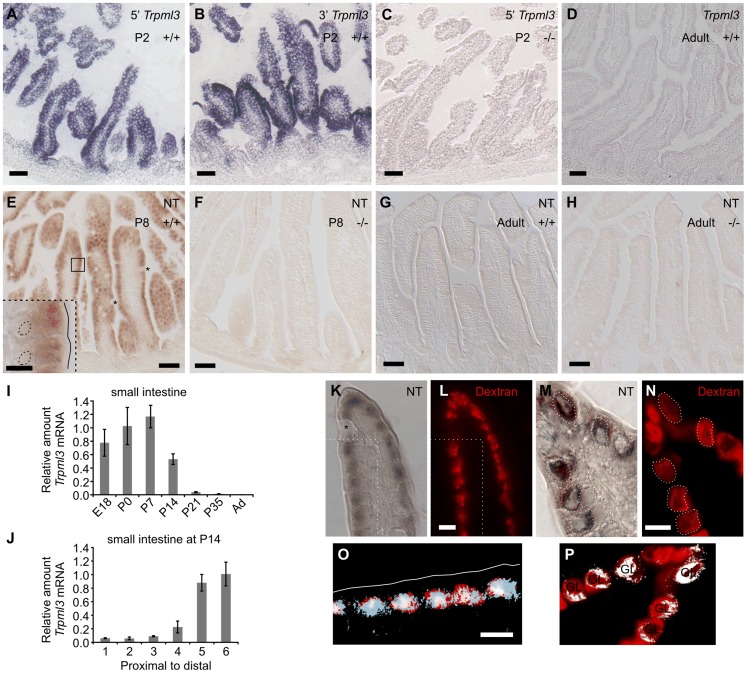
Intestinal enterocytes express *Trpml3* specifically during the suckling period and accumulate TRPML3 protein in their specialized endolysosomal organelles. (**A–D**) *In situ* hybridization (ISH) with two non-overlapping probes to *Trpml3* (complementary to 5′ and 3′ portions of its mRNA) reveals strong mRNA levels in (**A,B**) neonatal, but not (**D**) adult intestines. (**C**) Lack of hybridization on neonatal intestines of *Trpml3^−/−^* mice shows that the probe used specifically detects *Trpml3* mRNA. (**E–H**). Immunohistochemistry with an antibody to the N-terminus of TRPML3 (NT) on (**E,F**) neonatal and (**G,H**) adult intestines reveals that (**E**) neonatal but not (**G**) adult enterocytes express TRPML3 protein. Goblet cells (marked with asterisks) do not express TRPML3. (F,H) Lack of immunoreactivity in intestines from *Trpml3^−/−^* mice confirms that the immunoreactivity in wild type intestines specifically represents TRPML3 proteins. (**I–J**) RT-qPCR reveals that (**I**) the high levels of *Trpml3* mRNA in neonatal intestines subside by weaning, and that (**J**) by P14 distal intestine (i.e., ileum) of the suckling mouse, characterized by giant lysosomes, expresses higher levels of *Trpml3*. Each bar is the average of n = 3 experiments. Error bars represent the standard deviation. (**K,M**) Immunohistochemistry on P7 intestines in which prior exposure to Texas Red-dextran has labeled lysosomes (**L,N**). Enterocytes, but not goblet cells (labeled with an asterisk on **K**), are lysosome-rich and express TRPML3 protein. (**O, P**) Merging of both images (in **O**, only the region delimited with dotted lines in **K** and **L**). (**K,L,O**) In duodenal enterocytes, which contain multiple closely spaced lysosomes, the resolution of the anti-TRPML3 immunoreactivity does not discern individual lysosomes. However, the TRPML3-immunorreactivity occupies the same area of cytoplasm than the dextran-filled lysosomes, and not the portion of cytoplasm rich in endosomes closed to the apical membrane (outlined in white on **O**). (**M,N,P**) Illeal enterocytes contain a single giant lysosome (labeled GL on **P**), which is filled with dextran and immunoreacts with antibodies to TRPML3. Dotted lines in (**M**) outline the giant lysosome boundaries as defined by Texas Red-dextran in (**N**). Hence, lysosomes of enterocytes seem to contain TRPML3 protein. Scale bars are 50 µm except in the inset in E and in K–O, where they are 10 µm. Overall these data supports that TRPML3 protein localizes to lysosomes, although localization at nearby late endosomes is also possible.

While all major organs express *Trpml1* [14], [23], only a few cell types express the paralog *Trpml3*, including inner ear hair and marginal strial cells, olfactory and vomeronasal sensory neurons [15], [16] and melanocytes [17]. In order to determine the full expression profile of *Trpml3* in the mouse, we performed RNA in situ hybridization (ISH) on sagittal sections of newborn pups (postnatal days P1 and P2) and on sections of adult mouse organs, as well as quantitative RT-qPCR on a wide range of organs. We found expression of *Trpml3* in melanocytes of skin, principal cells of the kidney's collecting duct, alveolar macrophages of lung, choroid of the eye (probably retinal pigmented epithelial cells) and thymus (S1 Figure and AJC, NNR, TW and JGA, manuscript in preparation). While expression of *Trpml3* did not differ between neonates and adults for these cell types and organs, a notable exception was the epithelia of the intestinal villi, which expressed the highest levels of *Trpml3* mRNA in neonates but no detectable levels in adults (Fig. 1A–D). We confirmed that the neonatal in situ signal was specifically detecting *Trpml3* mRNA because it was obtained with two non-overlapping antisense probes (one complimentary to exons 1 to 5 and the other to exons 8 to 12; Fig. 1A,B) but not with control sense probes or with antisense probes in *Trpml3^−/−^* tissue (described below; Fig. 1C). However, the same antisense probes could not detect *Trpml3* mRNA in sections of adult intestine (Fig. 1D). We also performed immunohistochemistry (IHC) on intestines using antibodies raised against the N-terminus of mouse TRPML3 (TRPML3-NT) [15], which labeled the apical region of villus epithelial cells from neonatal (P8 and P7) *Trpml3^+/+^*, but not *Trpml3^−/−^*, mice (Fig.1E,F,K,M). Within intestinal villi of neonates, the cells expressing TRPML3 were the enterocytes, and not the secretory goblet cells or any cell within the lacteals (the internal portion of the villi, which is part of the lymphatic circulation; Fig. 1E,F,K,M). However, we could not detect TRPML3-NT immunoreactivity in sections of adult (P48) *Trpml3^+/+^* small intestine above the weak non-specific immunoreactivity levels of *Trpml3^−/−^* littermates (Fig. 1 G,H). We therefore conclude that neonatal, but not adult, enterocytes express *Trpml3* mRNA and TRPML3 protein.

Enterocytes live only for a few days, and those produced in neonates differ in many respects from those produced in adults. Neonatal enterocytes are specialized in the digestion of nutrients from suckled milk, and, as weaning approaches (∼P21 in the mouse), are replaced by “mature-feeding” enterocytes equipped for the digestion and absorption of nutrients from ingested chow [1], [6], [24]–[26]. Quantitative RT-PCR analysis of *Trpml3* mRNA levels in small intestine from prenatal (E18.5) to adult indicates that *Trpml3* mRNA levels peak during the first postnatal week (P7), subside as weaning approaches and reach undetectable levels in adults (Fig. 1I). By P14, *Trpml3* mRNA is more abundant in the distal (ileum) than proximal (duodenum) intestine (Fig. 1J), consistent with the spatiotemporal replacement of suckling enterocytes with mature enterocytes [1], [6], [24]–[26]. Hence, intestinal enterocytes express *Trpml3* during the postnatal period of suckling, but not afterwards.

Neither ISH nor IHC detected expression of *Trpml3* in the space between the villi, also called intervillus pockets and crypts, where the intestinal stem cells that produce the enterocytes reside [27] (Fig. 1A,B,E). Hence, it appears that mucolipin 3 acts in the postmitotic, differentiated enterocytes of suckling mice.

### TRPML3 protein localizes to the specialized endolysosomal organelles of suckling enterocytes

A defining characteristic of suckling enterocytes is that they do not uptake free amino acids from the intestinal lumen but instead endocytose proteins (and perhaps some fats) from the milk and deliver them to lysosomes for digestion [1], [3]. This requires specialized lysosomes which, in the more distal parts of the small intestine (ileum), form one giant lysosomal vacuole per enterocyte. We labeled lysosomes of neonatal mice by feeding them formula with Texas Red-conjugated dextran at P6 and examining its intracellular accumulation one day later (at P7; Fig. 1L,N). Immunohistochemistry with TRPML3-NT antibodies in sections of duodenum revealed immunoreactivity to the region of cytoplasm rich in lysosomes, and not in the more apical portion of cytoplasm that is richer in early endosomes (Fig. 1K,L,O). The high density of lysosomes in these duodenal enterocytes and the poor subcellular localization afforded by immunohistochemical amplification (DAB or tyramide amplification, the only means by which available antibodies can reveal the presence of TRPML3 protein) prevent us from unambiguously localizing TRPML3 to lysosomes (as opposed to adjacent organelles such as endosomes). In sections of ileum, where enterocytes display not a multitude of lysosomes but a single giant one, TRPML3 immunoreactivity also overlaps with the Dextran-filled giant lysosomal vacuoles (Fig. 1M,N,P).

Hence, endolysosomal TRPML3 channels localize to the specialized endolysosomal organelles (likely lysosomes, but perhaps also endosomes) of suckling enterocytes. This subcellular localization resembles that of other epithelial cells (LLC-PK1-CL4 and hair cells of the inner ear) in which TRPML3 localizes to intracellular vesicles, most of which co-express lysosomal markers [15], [16].

### During the postnatal period of suckling, intestines upregulate *Trpml1* but do not express *Trpml2*

**Figure 2:**
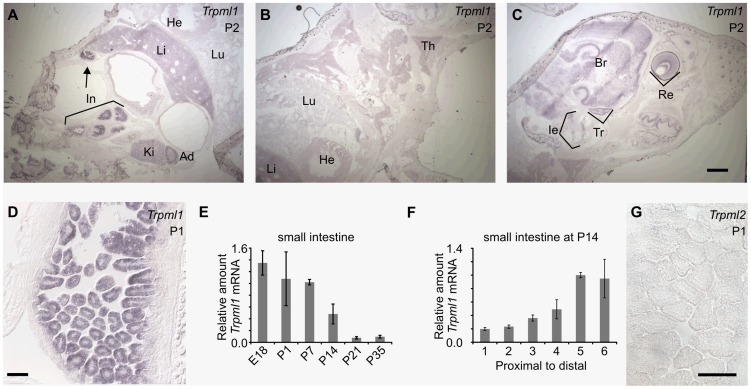
Neonatal intestines upregulate mRNA levels of mucolipin 1 (*Trpml1*), but do not express mucolipin 2 (*Trpml2*). (**A–D, G**) In situ hybridization (ISH) on sagittal sections of postnatal day 2 (A–C) and 1 (D,G) pups reveal (**A–D**) high expression of *Trpml1* mRNA in the epithelia of the intestinal villi but (**G**) lack of detectable *Trpml2* mRNA. In situ signal for Trpml1 mRNA reveal higher levels in neonatal intestines (In) than in any other organ including kidney (Ki), adrenal gland (Ad), liver (Li), lung (Lu), heart (He), thymus (Th), inner ear (Ie), trigeminal ganglia (Tr), brain (Br) and retina (Re). (**E**) RT-qPCR reveals that the levels of *Trpml1* mRNA on small intestine are high during suckling and subside to a much lower, baseline level by weaning. (**F**) RT-qPCR on equally divided segments of small intestine also reveals that by P14 *Trpml1* mRNA is more abundant in ileum (distal) than duodenum (proximal) and jejunum (middle). Normalized *Trpml1* levels are displayed relative to the level of *Trpml1* in proximal ileum at P14. Each bar is the average of n = 3 experiments. Error bars represent the standard deviation. Scale bars are 1 mm (A–C) or 50 µm (D,G).

We similarly examined the expression of the two other mucolipin genes, *Trpml1* and *Trpml2*, by ISH on neonatal mice. Although most cell types express *Trpml1* [14], [15], we noticed that in sections of neonates, intestines displayed higher levels of expression than all other organs and tissues (Fig. 2A–D). Quantitative RT-qPCR (Fig. 2E,F) revealed that the intestinal levels of *Trpml1* mRNA subsided by weaning and in a proximal-to-distal gradient, in a spatiotemporal pattern similar to that of *Trpml3* mRNA. The main difference between the two mucolipin genes is that *Trpml1* mRNA did not become undetectable in mature intestines, but stabilized at a lower level, as expected of a ubiquitously-expressed gene. Finally, *Trpml2* mRNA expression was restricted to some cells of thymus [28] but was undetectable in all other neonatal tissues, including the intestine (Fig. 2G). Hence, intestinal enterocytes upregulate *Trpml1* and *Trpml3* paralogs during suckling.

### Generation of mice null for *Trpml3* and for both *Trpml3* and *Trpml1*

**Figure 3:**
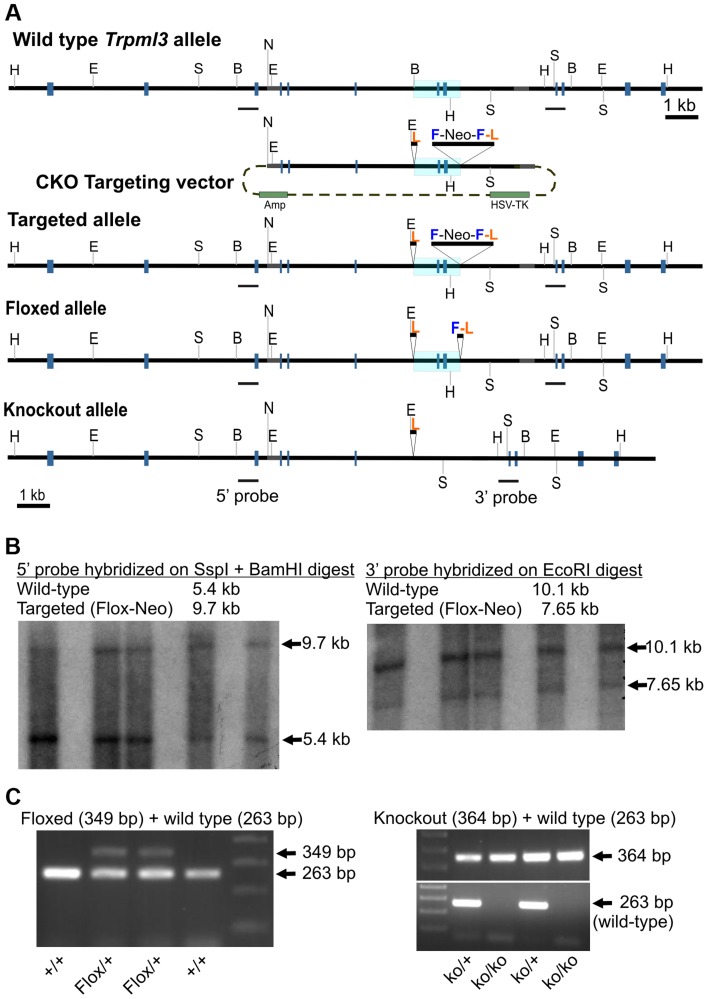
Generation of mice with knockout alleles of *Trpml3*. (**A**) Schematic representation of targeting construct and Wild type, Targeted (Flox-Neo), Floxed and Knockout alleles of *Trpml3*. The Mouse Genomics Informatics (MGI) name assigned to the KO allele is *Mcoln3^tm1.1Jga^*. Exons are represented by gray boxes and the area to be deleted is shaded in blue. Restriction enzymes indicated are *EcoR* I (E), *BamH* I (B), *Ssp I* (S), *Not* I (N) and *Hpa I* (H). *LoxP* sites are labeled L, *FRT* sites F, and Neomycin-expressing cassettes Neo. (**B**) Southern blots on genomic DNA from five ES cell clones demonstrating the presence of a targeted (Flox-Neo) allele of *Trpml3*, obtained by homologous recombination. Both 5′ and 3′ arms underwent homologous recombination. (**C**; left) PCR genotyping of a litter in which two mice carry the Floxed allele, created by expression of FLPe recombinase in mice bearing the Flox-Neo allele. (**C**; right) PCR genotyping of a litter in which mice carry one or both knockout alleles, created by expression of Cre recombinase in mice bearing the Flox-Neo allele.

In order to elucidate the role of mucolipins 3 and 1 in enterocytes during the suckling period, we engineered mice lacking functional *Trpml3*. We first generated mice bearing an allele in which *loxP* sites surrounded exons 7 and 8 of *Trpml3*, and then crossed them to mice expressing the Cre-recombinase ubiquitously prior to implantation (EIIa-Cre) in order to generate mice with a *Trpml3* mutant allele lacking exons 7 and 8 (termed *Mcoln3^tm1.1Jga^* by the Mouse Genomics Informatics but referred to as *Trpml3^−/−^* in this manuscript; Fig. 3). As confirmed by sequencing of cDNAs produced by this knockout allele, absence of exons 7 and 8 causes splicing from exon 6 to 9, generating a frameshifted mRNA with several stop codons and thus unable to synthesize transmembrane domains 2 to 6 and the channel pore [15]. In situ hybridizations with probes complimentary to exons upstream and downstream of the deletion were unable to detect *Trpml3* mRNA in neonatal intestines of *Trpml3^−/−^* mice, demonstrating that the mutated mRNA is unstable or produced at very low levels [15] (Fig. 1A–C). Furthermore, immunohistochemistry with antibodies to the amino or carboxyl ends of TRPML3 produced no immunoreactivity on sections of *Trpml3^−/−^* neonatal intestine (Fig. 1E,F), confirming that no detectable levels of TRPML3 protein, not even the truncated peptide encoded by exons upstream of the deletion, are produced by this allele. Hence, the deletion of exons 7 and 8 generates a complete knockout or null allele of *Trpml3*. The *Trpml3^−/−^* mice were born at the expected Mendelian ratios and were viable (of the 561 progeny obtained by mating *Trpml3^−/+^* mice, 23.5% were *Trpml3^−/−^* and 25.5% were *Trpml3^+/+^*; the percentage of born mice that died prior to weaning was 10.6% for *Trpml3^−/−^* and 9.9% for *Trpml3^+/+^*) and fertile.

Given the co-expression of mucolipins 3 and 1 in suckling enterocytes and the possibility that they may act redundantly (i.e., be able to replace one another), we also crossed *Trpml3^−/−^* and *Trpml1^−/−^* mice [12], [13] to generate *Trpml3^−/−^*;*Trpml1^−/−^* double knockouts (DKOs). These mutant mice were born and survived into maturity at the expected Mendelian ratios (of the progeny obtained by mating *Trpml3^−/−^*;*Trpml1^+/−^* mice that reached the weaning age of P21, 15 were *Trpml3^−/−^*;*Trpml1^−/−^*, 15 were *Trpml3^−/−^*;*Trpml1^+/+^*, and 29 were *Trpml3^−/−^*;*Trpml1^+/−^*), and they were fertile. Like *Trpml1^−/−^* mice, adult *Trpml3^−/−^*;*Trpml1^−/−^* mice had no overt phenotype until ∼6 to 8 months of age, when they developed ataxia due to the lack of mucolipin 1 [12], [13].

### Pathological vacuolation of neonatal enterocytes of mice lacking both mucolipins 3 and 1, but not of mice lacking a single mucolipin

**Figure 4:**
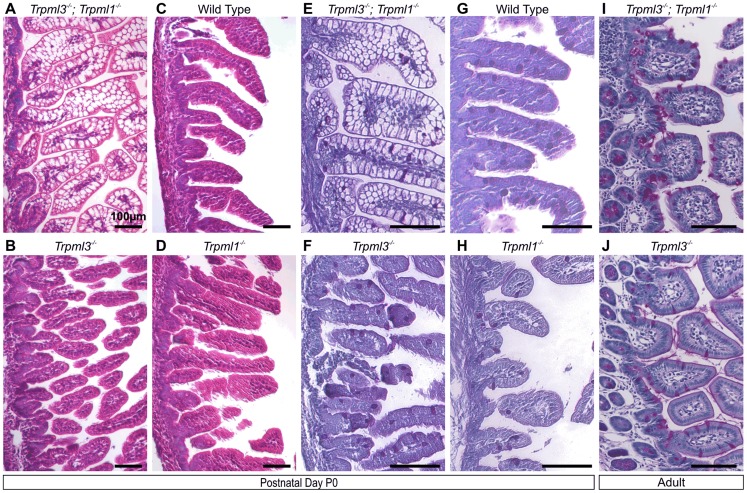
Pathological vacuolation of neonatal, but not adult, enterocytes lacking both mucolipins 3 and 1, but not either mucolipin alone. (**A–D**) Hematoxylin and eosin (H&E) staining of paraffin-embedded sections of intestine reveals abnormal vacuolation in (**A**) *Trpml3^−/−^;Trpml1^−/−^* pups, but not in (**C**) wild type, (**B**) *Trpml3^−/−^* or (**D**) *Trpml1^−/−^* pups. (**E,F**) Periodic acid-Schiff (PAS) staining of paraffin-embedded sections of neonatal intestines reveals that (**E**) the vacuolated intestinal cells of *Trpml3^−/−^;Trpml1^−/−^* mice are not mucin-filled, goblet cells (labeled red), which show a distribution undistinguishable from (**F**) *Trpml3^−/−^*, (**H**) *Trpml1^−/−^* and (**G**) wild type controls. (**I,J**) PAS staining of adult intestines reveals (**I**) no vacuolation of enterocytes from *Trpml3^−/−^;Trpml1^−/−^* mice and no other signs of pathology when compared with (**J**) *Trpml3^−/−^* littermate and wild type controls. All scale bars are 100 µm.

We histologically examined hematoxylin and eosin (H&E) stained paraffin sections of neonatal intestines from all the genotypes generated. While the intestines from *Trpml3^−/−^* and *Trpml1^−/−^* mice were undistinguishable from those of wild type littermates, the neonatal intestines of *Trpml3^−/−^*;*Trpml1^−/−^* mice were severely dysmorphic, comprised of cells with a vacuolated appearance (Fig. 4A–D and S2A–D Figure). In wild type intestines, an empty, vacuolated H&E appearance is characteristic of the mucus-secreting goblet cells. However, Periodic Acid-Schiff-staining, which labels the mucin-filled goblet cells, does not label the pathologically-vacuolated cells of neonatal *Trpml3^−/−^*;*Trpml1^−/−^* intestines and instead reveals a normal distribution of scattered goblet cells amidst the pathologically vacuolated enterocytes (Fig. 4E–H and S3C,G Figure). The normal appearance of neonatal enterocytes from *Trpml3^−/−^* and *Trpml1^−/−^* mice demonstrates that these two genes may substitute or compensate for one another and that, at least in the neonatal intestine, they can act redundantly (either by performing the exact same molecular function or by performing distinct roles that lead to the same or an equivalent outcome).

### Pathological vacuolation due to mucolipin co-deficiency is restricted to suckling, and not post-weaning, enterocytes

**Figure 5:**
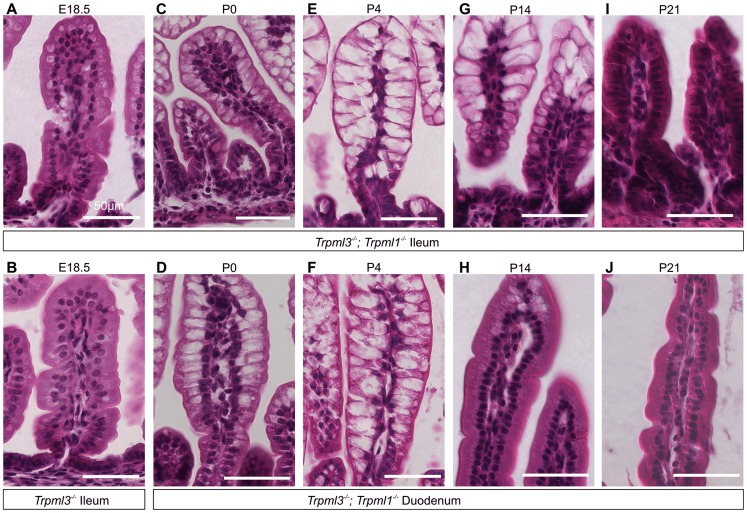
Pathological vacuolation of enterocytes from *Trpml3^−/−^;Trpml1^−/−^* mice is restricted to period of suckling, from birth to weaning. (**A,B**) H&E staining of distal ileum from embryos at 18.5 days post coitum (E18.5) reveals slight vacuolation in some illeal enterocytes of (**A**) *Trpml3^−/−^;Trpml1^−/−^* mice, but not of (**B**) *Trpml3^−/−^* mice. (**C–J**) H&E staining of (C,E,G,I) distal ileum and (D,F,H,J) proximal duodenum from *Trpml3^−/−^; Trpml1^−/−^* pups reveal (C,D) severe vacuolation by postnatal day 0 (P0), only several hours after birth. (**E,F**) Vacuolation is most severe by P4, and lasts throughout much of the period of suckling. At all time-points, intestinal enterocytes appear to develop a progressively enlarging vacuole as cells migrate from villus base to tip. (**H**) Sections from the proximal duodenum of P14 *Trpml3^−/−^;Trpml1^−/−^* mice show a nearly complete recovery, with some vacuolated enterocytes remaining at the villus tips. (**I,J**) By P21, intestinal enterocytes of the distal ileum and proximal duodenum from *Trpml3^−/−^; Trpml1^−/−^* mice are not vacuolated and appear normal. At no point do we observe vacuolation in aged-matched *Trpml3^−/−^*, *Trpml1^−/−^*, and wild type controls (Fig. 4 for P0, S2 Figure for P7). Scale bars are 50 µm.

Interestingly, adult *Trpml3^−/−^*;*Trpml1^−/−^* mice lacked pathologically-vacuolated enterocytes and their intestines had a normal appearance (Fig. 4I,J). Enterocytes arise from stem cells at the intestinal crypts, migrate for several days from the base towards the tip of the villi, and eventually are shed [6]. The intestine produces suckling enterocytes from late embryogenesis until ∼P12, when it starts producing adult like-enterocytes so that, by weaning (∼P21 in the mouse) none of the enterocytes are of the suckling type [1], [6], [24]–[26]. A time series on intestines of *Trpml3^−/−^; Trpml1^−/−^* mice reveals that enterocyte vacuolation is minimal in late embryos (Fig. 5A,B), but becomes pronounced after birth (Fig. 5C–F) and lasts until past P14, when it is present in the suckling enterocytes at the tip of the villi, but not in the newly-formed, mature enterocytes closer to the base (Fig. 5G,H). By weaning, none of the enterocytes are vacuolated (Fig. 5I,J). The vacuolated enterocytes show a gradient of severity along the villi that corresponds with their age (the length of time since differentiation; Fig. 5C–G): at the base, newly born enterocytes appear normal; at the tips, the oldest enterocytes appear the most vacuolated. As a result, the villi swell towards their tips and are dysmorphic. Hence, only the suckling enterocytes lacking mucolipins 3 and 1 suffer vacuolation, which emerges in nascent enterocytes and progresses as they age.

The vacuolated appearance of the neonatal enterocytes of *Trpml3^−/−^;Trpml1^−/−^* mice is strikingly similar to that of enterocytes from patients suffering abetalipoproteinemia [29] and mutant mice lacking apolipoprotein B [30], in which a deficiency in chylomicron formation results in massive lipid accumulation in the enterocytes. However, while an Oil Red-O staining reveals that the vacuoles of apolipoprotein B KO mice are loaded with fats [30], the same staining on neonatal *Trpml3^−/−^; Trpml1^−/−^* enterocytes reveals that their vacuoles are largely fat-free and that, overall, these intestines uptake fats from milk and secrete them into the lacteals as wild types do (S3 Figure). Hence, an intracellular accumulation of undigested fats is not the cause of vacuolation of enterocytes lacking mucolipins 3 and 1.

### Formation of pathological, membrane-bound organelles in neonatal enterocytes of *Trpml3^−/−^;Trpml1^−/−^* mice

**Figure 6:**
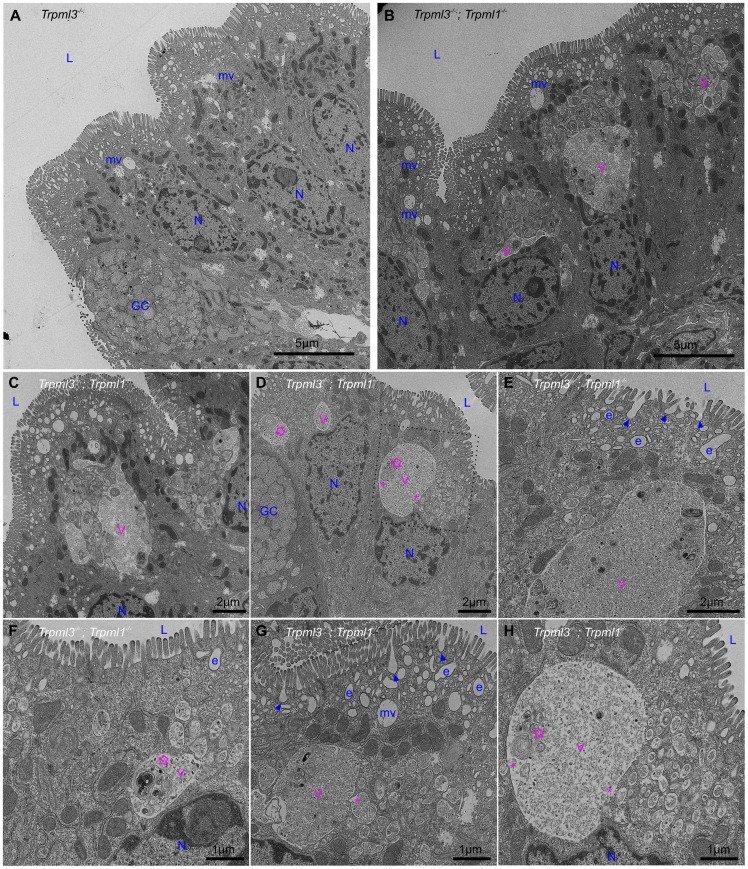
Formation of pathological vacuolar organelles in neonatal enterocytes lacking both mucolipins 1 and 3. Ultrastructural examination of ileum from (**A**) control *Trpml3^−/−^* and (**B–H**) *Trpml3^−/−^;Trpml1^−/−^* pups at P0. The intestinal epithelia of *Trpml3^−/−^;Trpml1^−/−^* mice contain normal appearing goblet cells (GC) and enterocyte nuclei (N). Their enterocytes display normally appearing nuclei (N), mitochondria and apical microvilli as well as the endocytic structures normally found at P0 (all labeled in blue and tentatively identified based on their ultrastructural features and subcellular location): invaginations presumed to be endocytic figures in the process of endocytosis (arrowheads), early endosomes (e) and late endosomes/multivesicular bodies (mv). However, *Trpml3^−/−^;Trpml1^−/−^* enterocytes also displayed abnormal organelles (labeled in pink), primarily a large vacuole (V) and/or multiple smaller vesicles, filled with granular material, some electron dense material and some multilamellar structures, often in the form of whorls of concentric membranes (empty arrows). There appears to be fusion (or fission) between some smaller vesicles and the large vacuole (asterisks). The dotted lines in (**D**) delineate the area magnified in (**H**). L, lumen of intestine.

In order to elucidate the subcellular basis of neonatal enterocyte vacuolation, we performed electron microscopy on neonatal intestines from *Trpml3^−/−^;Trpml1^−/−^* and their control littermates (*Trpml3^−/−^*, which do not vacuolate; Fig. 4B,F). Ultrastructural examination shortly after birth (Fig. 6) revealed that both genotypes displayed normal goblet cells as well as the normal brush border microvilli and apical endocytic machinery of enterocytes, with presumed endocytic figures (plasma membrane invaginations) between the microvilli, less apically-located endosomes and even deeper, multivesicular bodies (all organelle identifications are based on their ultrastructural appearance and thus tentative). However, the enterocytes of *Trpml3^−/−^;Trpml1^−/−^* neonates also displayed atypical organelles situated between the normal endocytic machinery and the nucleus. These pathological structures were membrane bound vacuoles, often a very large one next to multiple smaller ones, filled with some granular material and occasional multi-membranous lamellae (concentric rings of lipid membranes; empty arrows in Fig. 6D,F,H). Some of the smaller, pathological vesicles appear to be fusing (although they could be undergoing the reverse process of scission; asterisks in Fig. 6D,G,H) with the larger vacuole, which is typically located more basally within the enterocyte, closer to the nucleus.

**Figure 7:**
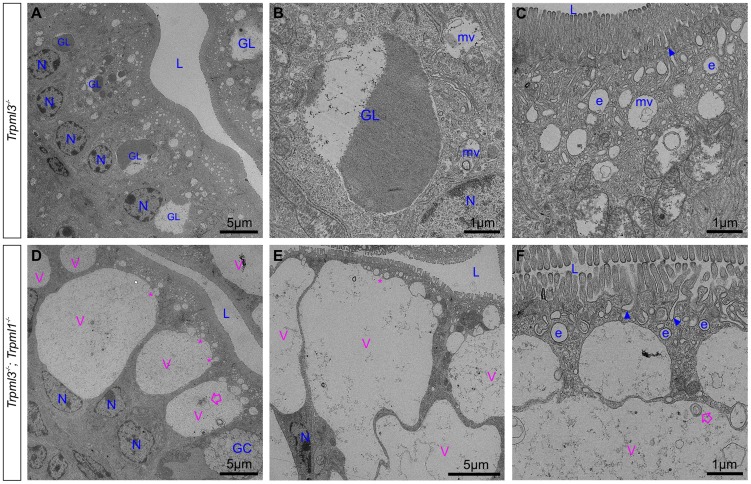
An enlarged, clear vacuole fills most of the intracellular space of *Trpml3^−/−^;Trpml1^−/−^* enterocytes by postnatal day 5. Ultrastructural examination of ileum from (**A–C**) control *Trpml3^−/−^* and (**D–F**) *Trpml3^−/−^;Trpml1^−/−^* littermates at P5. By this stage, ileal enterocytes of control *Trpml3^−/−^* pups contain their characteristic giant lysosome (GL), which is partially filled with electron dense material (presumably endocytosed milk proteins ready for intracellular degradation). By contrast, the pathological vacuoles (V, in pink) of *Trpml3^−/−^;Trpml1^−/−^* pups appear mostly empty, with very little electron-dense material, and are larger than at P0, occupying most of the cytoplasmic space. Smaller vesicles appear to be fusing with the larger vacuole (asterisks), which still contain some multilamellar, membranous whorls (empty arrows). Despite the aberrant deformation caused by the enlarged, pathological vacuoles, the enterocytes of *Trpml3^−/−^;Trpml1^−/−^* pups still display normal villi, endocytic figures in the apical membrane (arrowheads) and some early endosomes (e) (compare F with C). Additional abbreviations: L, lumen of intestine; GC, goblet cells; N, nuclei of enterocytes. Organelles present in control enterocytes are labeled in blue, whereas pathological structures are in pink.

By postnatal days 5–7, the enterocytes of control *Trpml3^−/−^*, *Trpml1^−/−^*, and wild type ileal enterocytes have the characteristic giant lysosome partially filled with electron dense material (presumably protein accumulated for digestion; Fig. 7A,B and S2B,C,E,F,H,I Figure). By contrast, by P5 and P7 ileal enterocytes of *Trpml3^−/−^;Trpml1^−/−^* pups lack this giant lysosome and instead have a greatly enlarged pathological vacuole that contains hardly any electron dense material but still has some membranous whorls (empty arrows on Fig. 7D,F and S2K,L Figure). These pathological vacuoles appear to be fusing at their apical extreme with presumed endosomes (asterisks in Fig. 7D,E) and become larger as the enterocytes age and reach the tip of the villi (Fig. 7D), causing the deformation and enlargement of the entire enterocyte. Despite their deformity, vacuolated *Trpml3^−/−^;Trpml1^−/−^ enterocytes* still display their characteristic brush border microvilli, apical plasma membrane invaginations and endosomes (Fig. 7C,F). Hence, ultrastructural examination of suckling-type enterocytes from *Trpml3^−/−^;Trpml1^−/−^* mice reveals that their vacuolated appearance apparent with H&E histology results from the formation of large, vacuolar organelles of an abnormal nature (i.e., never seen in control enterocytes) that form instead of (and in the same subcellular location as) the giant lysosomes characteristic of wild type ileal enterocytes.

### The pathological vacuoles of suckling enterocytes lacking mucolipins 1 and 3 are aberrant hybrid organelles of fused endosomes and lysosomes

**Figure 8:**
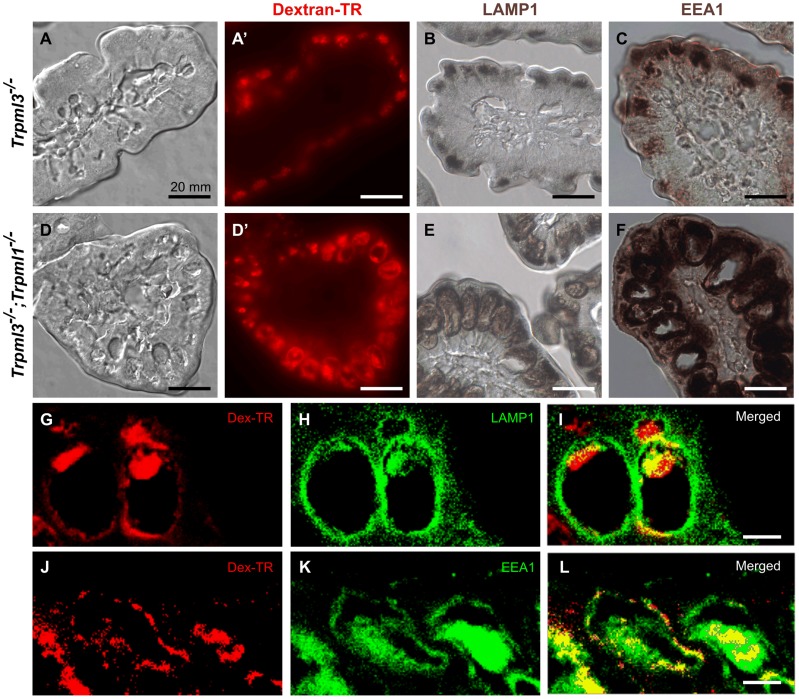
The pathological vacuoles of suckling enterocytes lacking mucolipins 1 and 3 are aberrant hybrid organelles with endosomal and lysosomal components. Cryosections of intestines from 3 hour old (**A–C**) control *Trpml3^−/−^* and (**D–F**) *Trpml3^−/−^;Trpml1^−/−^* littermates that had been fed formula with Texad Red-dextran immediately after birth. (**A,A′,D,D′**) Nomarski (**A,D**) and fluorescent (**A′,D′**) images of the same sections reveal that neonatal enterocytes endocytose the ingested Texas Red-dextran, which accumulates in the pathological vacuoles of *Trpml3^−/−^;Trpml1^−/−^* enterocytes (**D′**). Immunohistochemical staining with antibodies to the lysosomal marker LAMP1 (B,E) and to the early endosomal marker EEA1 (C,F) reveal that the pathological vacuoles of *Trpml3^−/−^;Trpml1^−/−^* enterocytes contain endosomal and lysosomal components and thus may result from the fusion of both types of organelles. (**G–L**) Confocal optical sections of immunofluorescent staining for LAMP1 (G–I) or EEA1 (J–L) on *Trpml3^−/−^;Trpml1^−/−^* intestines from the same P0 animal used in (D–F) reveal that endocytosed dextran accumulates in the pathological vacuoles, which contain both LAMP1 and EEA1. Due to the high level of neonatal enterocytes autofluorescence, which spans most of the detectable optical spectrum, secondary antibodies were conjugated with DyLight 405 and the 405 nm signals are pseudocolored green. Scale bars are 10 mm (A–F) or 20 µm (G–L).

In order to elucidate how this pathological vacuole forms, we fed formula with Texas Red-dextran to pups immediately after birth and examined their intestines three hours later. At this point, enterocytes have endocytosed the dextran which, in control mice, accumulates in the lysosomes as it is indigestible (Fig. 8A,A′). On the other hand, in enterocytes from *Trpml3^−/−^;Trpml1^−/−^* mice the dextran accumulates in the pathological vacuole, demonstrating that it accumulates endocytosed cargo normally destined to lysosomes (Fig. 8D,D′).

**Figure 9:**
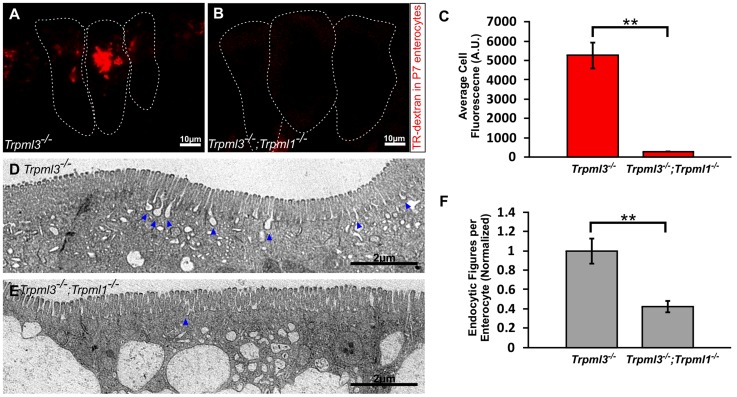
Reduced endocytotic rate in P7 neonates lacking mucolipins 3 and 1. (**A–B**) Confocal images of enterocytes from P7 littermates fed Texas Red-Dextran 120 minutes prior to tissue collection. There is an accumulation of dextran in *Trpml3^−/−^* enterocytes (A) whereas there is minimal dextran accumulation in *Trpml3^−/−^;Trpml1^−/−^* enterocytes (B). (**C**) Average cellular levels of endocytosed dextran, calculated from 3 pups per genotype. The quantification reveals a significant reduction of dextran uptake in *Trpml3^−/−^;Trpml1^−/−^* enterocytes compared to *Trpml3^−/−^* enterocytes (p = 0.002). For each animal, the value used is an average from 10 to 20 enterocytes. (**D–E**) Representative electron micrographs showing the apical membrane of enterocytes from P7 *Trpml3^−/−^* (D) and *Trpml3^−/−^;Trpml1^−/−^* littermates (E) where endocytic figures are in the process of endocytosis (arrowheads). (**F**) Average apical-membrane endocytic figures observed in electron micrographs of *Trpml3^−/−^;Trpml1^−/−^* pups is significantly lower than that observed in *Trpml3^−/−^* littermates (p = 0.006). Three pups per genotypes were used for this analysis. For both experiments, error bars indicate SEM. P value was calculated with a Student's t test.

An enlarged cytoplasmic vacuole accumulating endocytosed materials could result from an overall increase in endocytosis and/or a decrease in exocytosis, as both would increase the amount of intracellular organelle membrane. We looked for alterations in these processes in three separate ways (S4 Figure). First, we assessed transcytosis, by which maternally-provided antibodies are endocytosed from the lumen of the intestine into the suckling enterocytes and the delivered by exocytosis at the basolateral membranes to the lymphatic circulation [31]. We fed pups with biotinylated mouse IgGs diluted in infant formula and, as controls, with formula alone or with biotinylated chicken IgY (which are not recognized by mouse FC receptors and thus not internalized by mouse suckling enterocytes). We used ELISA to measure the levels of transcytosed biotinylated antibodies and found that, as expected, wild type pups transcytosed mouse IgG, but not chicken IgY (S4E Figure). However, we also found that, despite vacuolation of their enterocytes, *Trpml3^−/−^;Trpml1^−/−^* pups transcytosed the same amount of mouse IgG as wild type or *Trpml3^−/−^* pups (S4E Figure). Because an increase in endocytosis or a decrease in exocytosis would affect the amount of transcytosed IgG, our results suggest that neither process is altered by mucolipin co-deficiency. Second, we also assessed non-receptor mediated endocytosis by quantifying the amount of Texas Red-conjugated dextran that entered duodenal enterocytes of neonatal pups upon feeding. Despite the different subcellular distribution of endocytosed dextran (in a pathologically large vacuole in *Trpml3^−/−^; Trpml1^−/−^* enterocytes versus multiple lysosomes in enterocytes of *Trpml3^−/−^* control littermates), the average net amount per enterocyte did not differ with genotype and subcellular pathology (S4A–C Figure). Finally, we also quantified the number of plasma membrane invaginations which represent endocytotic or exocytotic events (see examples in figs. 7C,F), from suckling enterocytes at P0 (S4D Figure). We found that, despite the incipient intracellular vacuolation of *Trpml3^−/−^;Trpml1^−/−^* enterocytes, the number invaginations at their plasma membrane did not differ from that of control littermates. It is important to note that we assessed endocytosis immediately after birth, when the pathological vacuoles are forming, since our aim is to determine whether increased endocytosis is the cause of vacuolation, rather than a result of it (which we address below, in Fig. 9). Altogether, we find no evidence for an increased rate of endocytosis, or a decreased rate of exocytosis, that might account for the endolysosomal vacuolar enlargement of *Trpml3^−/−^;Trpml1^−/−^* enterocytes. This must occur due to a deficiency in intracellular membrane transports, which is consistent with the presumed localization of mucolipins to the membranes of lysosomes and/or endosomes.

Two proposed roles for mucolipin channels are to (1) release calcium from lysosomes to trigger their fusion with endosomes (or vice versa) and (2) once fused into a transient hybrid organelle, release calcium from it to trigger scission and thus reformation of lysosomes and endosomes [7], [32]–[38]. In the first case, we would expect that the enlarged pathological vacuole of *Trpml3^−/−^; Trpml1^−/−^* enterocytes, filled with endocytosed material, would be an enlarged endosome or lysosome resulting from a block in fusion between these organelles. In the second case, we would expect that the enlarged pathological vacuole would be a hybrid organelle resulting from the continuous fusion of endosomes with lysosomes without the subsequent scission. By labeling with antibodies to the lysosomal marker LAMP1 and the early endosome marker EEA1 we found that the pathological vacuole of *Trpml3^−/−^;Trpml1^−/−^* enterocytes have both lysosomal and endosomal components (Fig. 8B,C,E,F). Such a pathologically enlarged endolysosomal hybrid organelle would not result from the proposed block in fusion between endosomes of lysosomes, but instead from an increase in the rate of fusion or, as previously proposed [7], [33]–[35], [39], [40], a subsequent decrease in the rate of scission (but see discussion below for potential caveats and alternatives to these interpretations).

### Diminished endocytosis from the intestinal lumen in vacuolated enterocytes

The normal rates of endocytosis immediately after birth, when vacuoles are forming (as seen in figs. 6B–H), suggest that an increase in endocytosis is not the cause for vacuolation (S4 Figure). However, the severe vacuolation reached hours to days later, when vacuoles occupy the vast majority of the enterocyte cytosol (Figs. 5, 7D–E, and S3J–L Figure), might to interfere with the endocytic process. To test this hypothesis we assessed endocytosis at postnatal day P7, when enterocyte vacuolation is severe (Fig. 9). First, we fed Texas Red Dextran to pups by mouth, so that it would travel to the intestinal lumen where, as happens to ingested nutrients, it will be uptaken by endocytosis at the apical membrane of enterocytes. We found that the amount of luminal dextran endocytosed by enterocytes of *Trpml3^−/−^;Trpml1^−/−^* pups was greatly diminished compared to that of control (*Trpml3^−/−^*) littermates (Fig. 9A–C). Second, we also quantified the density of endocytic figures (invaginations) at the apical plasma membrane of enterocytes, and again detected a great reduction in *Trpml3^−/−^; Trpml1^−/−^* pups compared to of control (*Trpml3^−/−^*) littermates (Fig. 9D–F). Hence, severely vacuolated enterocytes of *Trpml3^−/−^; Trpml1^−/−^* endocytose materials from the intestinal lumen at a greatly reduced rate. Given that nutrients such as proteins are uptaken by enterocytes from the intestinal lumen by apical endocytosis, we next predicted there would be a nutritional deficit in *Trpml3^−/−^;Trpml1^−/−^* pups.

### *Trpml3^−/−^;Trpml1^−/−^* mice fail to thrive due to reduced growth during suckling and recover after weaning

**Figure 10:**
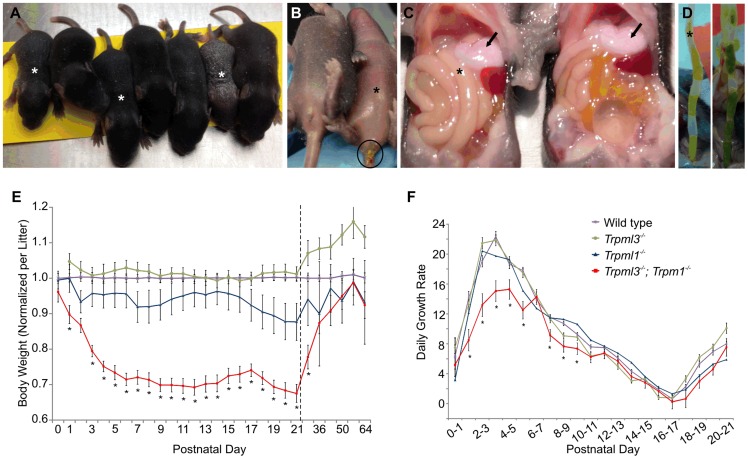
Failure to thrive of suckling mice lacking mucolipins 3 and 1. (**A–D**) *Trpml3^−/−^;Trpml1^−/−^* pups, denoted by asterisks, shown next to their *Trpml3^−/−^* control littermates. (**A**) A litter at P8, in which the three smallest pups have the *Trpml3^−/−^;Trpml1^−/−^* genotype. (**B**) Ventral view of a pair of littermates at P7, of which the one lacking mucolipins 3 and 1 is smaller and suffers diarrhea (circled). (**C**) Viscera of a pair of littermates at P7 shows that the pup lacking mucolipins 3 and 1 has the stomach filled with milk (arrows), which demonstrates its ability to suckle and ingest milk. (**D**) Extended distal intestines of a pair of littermates at P10 demonstrates diarrhea (liquid feces) of the *Trpml3^−/−^;Trpml1^−/−^* pup (left), compared with the pelleted feces of its control littermate (right). (**E**) Body weights of mice lacking mucolipins 1, 3 or both, normalized to the weights of wild type and heterozygote littermates, reveals that *Trpml3^−/−^;Trpml1^−/−^* mice become comparatively smaller after birth but partially recover after weaning (which occurs at P21, as indicated by the dashed line). Error bars denote SEM. Sample sizes (number of mice per genotype at each age) are n = 26 to 90 for wild type, n = 10 to 13 for *Trpml3^−/−^*, n = 7 to 16 for *Trpml1^−/−^* and n = 12 to 28 for *Trpml3^−/−^;Trpml1^−/−^*. Asterisks denote statistically different weights between *Trpml3^−/−^;Trpml1^−/−^* and *Trpml1^−/−^* mice (Student's t-test, p<0.001 except at P2, with p<0.05, and P29, with p<0.01). (**F**) Daily growth rates reveal that the lower weight of *Trpml3^−/−^;Trpml1^−/−^* pups is not due to weight loss but to reduced growth prior to postnatal day 10. Error bars denote SEM. Sample sizes (number of pups per genotype at each age) are n = 23 to 109 for wild type, n = 20 to 78 for *Trpml3^−/−^*, n = 32 to 66 for *Trpml1^−/−^* and n = 8 to 28 for *Trpml3^−/−^;Trpml1^−/−^*. Asterisks denote statistically different rates of growth between *Trpml3^−/−^;Trpml1^−/−^* and all other mice (Student's t-test, p<0.01 except at day 7 to 8, with p<0.05).

Enterocytes are the nutrient absorbing cells of intestine, and thus their severe dysmorphism and impaired endocytosis from the intestinal lumen in suckling mice lacking mucolipins 3 and 1 would be expected to cause malnourishment and affect growth. We did notice that *Trpml3^−/−^;Trpml1^−/−^* pups were smaller than those of *Trpml3^−/−^*, *Trpml1^−/−^* and wild type littermates (Fig. 10A,B). These smaller pups displayed normal suckling behavior and their stomachs were filled with milk (Fig. 10C), indicating that their reduced size was not due to a diminished intake of milk. Furthermore, they had diarrhea (Fig. 10B,D), as would be expected from a defect in intestinal absorption.

We then measured the weights of *Trpml3^−/−^;Trpml1^−/−^*, *Trpml3^−/−^*, *Trpml1^−/−^* and wild type littermates from birth (P0) until maturity (P64). By comparing the weights of each mutant relative to that of wild types (Fig. 10E), we found that while mice of all genotypes displayed similar weights at birth, those lacking both mucolipins (but not those lacking only mucolipin 3 or mucolipin 1), became relatively smaller during their first five days of life, reaching ∼70% of the weight of wild type mice. This weight differential was maintained during the entire period of suckling (until P21). However, after weaning, when normal mature enterocytes have replaced their vacuolated enterocytes of the suckling period, the weight of *Trpml3^−/−^;Trpml1^−/−^* mice slowly recovered, reaching weights similar to those of wild types by 7 to 9 weeks of age, as young adults (Fig. 10E). We also measured the percent daily growth during suckling and found that the failure to thrive of *Trpml3^−/−^;Trpml1^−/−^* pups was not due to a loss of weight at any time, since they grew every day, but to a diminished growth rate during the first half of the suckling period (Fig. 10F). This reduction in the rate of growth was most pronounced during the first 6 days of life. Hence, suckling mice lacking mucolipins 3 and 1 can, despite their severe enterocytic pathology, absorb sufficient nutrients from maternal milk to sustain continuous growth, but they do so at a reduced rate when compared to control mice.

## Discussion

The subcellular defects of mucolipidosis type IV (MLIV) are quite diverse and vary among cell types. While neurons accumulate electron dense bodies with glycosaminoglycans and lipids, epithelial cells tend to display electron lucent vacuoles with a varying amount of fribrillogranullar material and scattered multilamellar membranous whorls [41]–[45], very similar to the vacuoles of neonatal enterocytes lacking mucolipins 1 and 3 (Figs. 6H, 7D–F). The lack of staining of neonatal *Trpml3^−/−^; Trpml1^−/−^* enterocytes (S3 Figure and Fig. 4E) with Periodic Acid Schiff, which labels glycosaminoglycans, with Toluidine Blue, which labels polysaccharides and nucleic acids, and with Oil Red O, which labels lipids, confirm that their pathological vacuoles do not densely accumulate these substances, which are found in the inclusion bodies of neurons from MLIV patients. However, what is common to the pathology of all types of cells from MLIV patients is the concentric whorls of membrane, which we also see in *Trpml3^−/−^;Trpml1^−/−^* enterocytes. One important difference is that, while symptoms take months to develop in MLIV patients and mice lacking mucolipin 1 [12], [46], the phenotype of mice lacking both mucolipins develops immediately after birth, as their enterocytes severely vacuolate within hours. Hence, these double mutant cells seem to experience a greatly accelerated form of mucolipidosis type IV.

The slow onset of MLIV symptoms pose difficulties in elucidating the subcellular mechanism by which they form and hence in elucidating the subcellular function of mucolipin 1. Our study of neonatal enterocytes lacking both mucolipins offers the advantages of (1) eliminating genetic redundancy between the two mucolipins co-expressed by these cells and (2) examining the effects in a cell type that generates *de novo* an elaborate endolysosomal system [5]. Multiple roles have been proposed for the function of mucolipins in various cell types, either mammalian TRPML1, mammalian TRPML3, or orthologs in other species such as CUP-5 in nematodes, TRPML in flies and TRP-ML in amoebas [7], [32]–[38], [47]–[50]. These are letting calcium out of endosomes and lysosomes in order to: (1) regulate endocytosis and/or exocytosis, (2) favor the counterionic entry of protons to acidify the lysosomal lumen and thus allow hydrolytic activity, (3) promote the fusion with autophagosomes and endosomes and (4) facilitate the scission of the resulting endolysosomal hybrid organelle to reform endosomes and lysosomes. Our results in newborn enterocytes rule out defects in endocytosis or exocytosis (S4 Figure) and are not consistent with a defect in lysosomal degradation, since the pathological vacuoles appear largely empty despite their apparent fusion with endosomes and their collection of endocytosed materials (Figs. 7D–F and 8). The presence of both endosomal and lysosomal markers in the enlarging pathological vacuoles (Fig. 8E,F), the accumulation of endocytosed material normally destined to lysosomes in this pathological vacuoles (Fig. 8D,D′) and the fusion-looking figures observed ultrastructurally between apical endosomes and the apical side of the pathological vacuoles (Fig. 7D,E) are also incompatible with a deficiency in endolysosomal fusion. Instead, all these observations suggest a role of mucolipins in the scission required to disassemble the normally transient hybrid organelles into smaller endosomes and lysosomes. Alternatively, these vacuoles could arise by an increase –rather than a decrease- in the rate of endolysosomal fusion, although that would imply a role of mucolipins in preventing, rather than facilitating, vesicle fusion, a role for which there is no support in the published literature. A block of scission or an increase (but not decrease) in fusion would generate the enlarged endolysosomal vacuoles we observe in neonatal enterocytes. However, it must be noted that the pathological vacuoles of *Trpml3^−/−^;Trpml1^−/−^* enterocytes are aberrant organelles unlike the hybrid organelles that normally form from the transient fusion of late endosomes and lysosomes, which may not (though this is not known for enterocytes) incorporate the early endosome marker EEA1. For this and other reasons, definitive proof that increased fusion, decreased scission or another subcellular abnormality cause vacuolation is wanting but beyond the scope of this study, which is limited by the inability of neonatal enterocytes to be cultured and thus for the direct observation of their vesicle dynamics.

The upregulation of mucolipin 1 and selective expression of mucolipin 3 in suckling enterocytes is in keeping with the high activity of endolysosomal system in these cells, which use these organelles to uptake and digest nutrients from milk [1], [3], [4]. Interestingly, embryonic enterocytes of nematodes also express the mucolipin CUP-5, which they require for the endolysosomal uptake and digestion of yolk [51]. Furthermore, zebrafish orthologs of TRPML1 and TRPML3 are expressed highly (Mcoln1a) or exclusively (Mcoln3b) in the yolk syncytial layer or periblast [52], [53], the lysosome-rich organ used by fish tadpoles for the uptake and digestion of yolk [54], [55]. Hence, we propose that a mucolipin-endowed endolysosomal system in enterocytes or other specialized nutrient absorbing cells of the young plays an evolutionarily conserved role in the intracellular digestion of maternally-provided nutrients, whether milk in mammals or yolk in oviparous species.

The specializations of neonatal enterocytes for the absorption of milk nutrients are critical for neonatal growth and survival. A mutation in the Blimp1 transcriptional repressor, normally expressed in enterocytes until the suckling to weaning transition, results in only mature-like enterocytes and, due to their inability to properly digest milk, growth retardation (failure to thrive) and neonatal mortality [25], [26]. It may therefore come as a surprise that *Trpml3^−/−^;Trpml1^−/−^* pups, with their severe enterocyte vacuolation, are fully viable and at all times able to grow, even if at a reduced rate. Several observations may explain why their failure to thrive is partial. First, although proteins in milk are digested in lysosomes, sugars and many fats are not. Lactose, the main sugar in milk, is digested by lactase enzymes at the brush border microvilli membranes into glucose and galactose, which are then absorbed via plasma membrane transporters. The normal microvilli of vacuolated *Trpml3^−/−^;Trpml1^−/−^* enterocytes (Figs. 6 and 7) suggest that they would be able to digest and absorb sugars. Milk fats are largely digested by extracellular lipases in the lumen of the digestive track, taken up by enterocytes for assembly into chylomicrons in the Golgi apparatus and then secreted into the lacteals and hence the lymphatic circulation [56], [57]. The process does not involve the specialized lysosomes of suckling enterocytes. Indeed, the Oil Red O staining revealed a distribution of fat droplets in the lacteals of *Trpml3^−/−^;Trpml1^−/−^* villi, despite the severe vacuolation of their enterocytes (S3 Figure). Nonetheless, a complete inability of milk protein digestion might be expected to cause lethality or at least cause weight loss. However, because enterocytes are constantly generated and the newly differentiated ones, situated at the base of the villi, are not vacuolated (Fig. 5), they should be able to endocytose and digest milk proteins. Even the older, vacuolated enterocytes may be able to digest the limited amounts of milk proteins that have been taken up by endocytosis, as their enlarged vacuoles appeared mostly empty (Fig. 7D–F and S3 Figure). Instead, the diarrhea of *Trpml3^−/−^;Trpml1^−/−^* pups (Fig. 10B,D) as well as the deficiency of their vacuolated enterocytes in endocytosis across the apical plasma membrane (Fig. 9) both suggest a defect in the uptake of proteins from the intestinal lumen. Such a defect can explain their partial failure to thrive, which is characterized by reduced growth but not weight loss or lethality. Finally, the replacement of enterocytes around weaning by mature enterocytes that do not co-express mucolipins and do not vacuolate in their co-absence explains the post weaning growth recovery of *Trpml3^−/−^; Trpml1^−/−^* mice.

Our results clearly demonstrate that, at least in suckling enterocytes, mucolipins 3 and 1 can replace one another. This redundancy may not explain the delayed onset of symptoms observed in MLIV patients and *Trpml1^−/−^* mice, because most of the affected cell types (such as neurons and corneal epithelial cells) do not express TRPML3 [15] (AJC, NNR, TW and JGA, manuscript in preparation), and in the absence of TRPML1 they do not upregulate TRPML3 as a compensatory mechanism [58]. However, a potential therapeutic implication of the ability of TRPML3 to substitute for TRPML1 is that ectopic expression of the endogenous *Trpml3* gene of MLIV patients, provided the technology were developed, could prevent their symptoms.

Another implication of our results to MLIV, and to lysosomal disorders in general, is that certain nutrients provided by maternal milk are not as much used as energy sources as they are as constituents of the growing brain and retina to properly develop and function [56], [57]. Hence, some of the neurological and retinal symptoms of MLIV and other lysosomal storage diseases might result from, or at least be aggravated by, defective absorption of milk nutrients during suckling. We suggest that nutrient absorption during suckling be examined for patients with lysosomal disorders, as any potential malnutrition might be avoided with, for example, amino acid-based formula that lacks proteins or parenteral nutrition.

Furthermore, the role of mucolipins extends beyond mucolipidosys type IV to other lysosomal storage disorders, such as Nieman-Pick disease, as it has been reported that the accumulation of luminal lipids in the cells of these patients block mucolipins and aggravate the symptoms [21], [22]. Hence, it would be advisable to examine the intestines of newborns with these diseases for pathological signs like those here described. These signs would not be detected after weaning but could have long-term, yet avoidable, effects in the patients.

On the converse, the failure to thrive we have found in mice lacking mucolipins 3 and 1 point to the importance of examining these and other endolysosomal system proteins, as well as the genes encoding them, in patients with intractable, neonatal failure to thrive and diarrhea, one of the most common causes of infant mortality worldwide (http://www.who.int/gho/child_health/mortality/en/index.html).

## Methods

### Ethics

All animal care and procedures were in strict accordance with the *Guide for the Care and Use of Laboratory Animals* published by the National Institutes of Health and were approved by the Northwestern University Institutional Animal Care and Use Committee.

### Animals

Mice were housed in the barrier rooms of Northwestern University's animal facility. We obtained tissues from either CD1 mice (Charles River), from *Trpml3^−/−^* and *Trpml3^+/+^* littermates with a genetic background ∼75% C57BL/6 and ∼25% Sv129/Ola [15], from *Trpml1^−/−^* and *Trpml1^+/+^* littermates with a genetic background ∼75% C57BL/6 and ∼25% 129S6 [12] and from *Trpml3^−/−^;Trpml1^−/−^*, and littermates with a genetic background of ∼75% C57BL/6, ∼12.5% Sv129/Ola and ∼12.5% 129S6.

### Generation of *Trpml3* knockout mice

To begin generation of the *Trpml3* targeting construct we electroporated mouse genomic DNA (BAC ID: RP24-2767M11), containing the *Trpml3* gene, into SW106 electrocompetent bacterial cells. We then inserted into the plasmid pL253 two PCR amplified ‘mini-arms’ of 415 bp and 461 bp genomic sequence corresponding to portions of *Trpml3* introns 3 and 8, respectively. The designed primers included restriction sites to ligate mini-arms in pL253 (5′miniarm: (F) 5′-TAGCGGCCGCACAGAATGAGTTCCAGG ACAGCC-3′, (R) 5′-ACTCGAGCTCCAGGCGTTCCAAGGTGG-3′; and 3′miniarm: (F) 5′-ATCAAGCTT AAGGCATCCTGTTTGAGCAGCC-3′, (R) 5′-CTACTAGTCGAACAGACTGCCAGCAGAGG-3′) and thus generate the first phase of the targeting construct (construct#1). Co-electroporation of BAC DNA and construct #1, linearized at *Hind* III site, allowed for gap repair retrieval of 8.1-kb of *Trpml3* genomic sequence, including *Trpml3* exons 4–8, via homologous recombination in SW106 cells (construct #2). To insert the 5′ loxP site upstream of exon 7, we PCR amplified a fragment of plasmid pL452, containing a *loxP*-*Neo* cassette-*loxP* sequence, using 100 bp long primers designed with 50 bp homology to the desired location within intron 6: (F) 5′-CAAAGTGGGCTAAGCATGGCAGTTTCGTGGGGGCTGTTGTGAGAATTC ATAACTTCGTATAATGTATGCTATACGAAGTTATCGACCTGCAGCCTGTTGA-3′ and (R) 5′-CTCAGC ACACTTCCCACCTGACTCTATTCCACGCACTTTGTGTGTACAATAACTTCGTATAGCATACATTATACGAAGTTATGTCGAGGCTGATCAGCGA-3′. We electroporated the PCR fragment with construct#2 to obtain the *Trpml3* genomic sequence with a *loxP*-*Neo* cassette-*loxP* insertion upstream of exon 7 (construct #3). To excise the *Neo* cassette and leave a single *loxP* site remaining, we transfected construct#3 into SW106 cells, previously induced to express *Cre* recombinase by growth in 10% arabinose media for 1 hour (construct #4). To insert a loxP site downstream of exon 8 and obtain the final targeting construct. we PCR amplified sequence from plasmid pL451, containing a *FRT*-*Neo* cassette-*FRT-loxP* sequence, using 100 bp long primers designed with 50 bp homology to the desired location within intron 8: (F) 5′-GTCTAGGAGCTCTTTCTCACAGCTCTGCCTGCCTCTGCCTCCTGGGTGGAAGTTC CTATTCTCTAGAAAGTATAGGAACTTCAGGTCTGAAGAGGAGTTT-3′ and (R) 5′-TGAGAGACTTCT AGTAGCTGAGTGGTGGTGGTGTATGCCTTTAATCCTACATAACTTCGTATAGCATAACATTATACGAAGTTATATTATGTACCTGACTG-3′ (construct #5). The targeting construct was linearized with *Not* I and Northwestern's TTML facility transfected it into embryonic stem (ES) cells with a HM1/129Ola genetic background and selected for recombined ES cells with a *Neo* cassette by growing in neomycin-containing media. Correctly targeted ES cell clones from 96-well plates were screened and identified by PCR,, clonally expanded and further screened by Southern blot to demonstrate correct 5′ and 3′ homologous recombination. Two ES cell clones carrying a correctly targeted *Trpml3* allele were injected into E3.5 C57BL/6 blastocysts and transferred into the uterine horns of pseudopregnant (E2.5) females. Male chimeric mice were crossed to C57BL/6 females. Germline transmission was evidenced by coat color and was confirmed by PCR. The *Trpml3^+/Flox^* progeny were then crossed to the B6.FVB-TgN(EIIa-Cre)C5379Lmgd transgenic mouse (Jackson stock #003724) expressing *Cre* recombinase in all cells of early (pre-implantation) embryos under control of the adenovirus EIIa promoter, to excise the *Trpml3* (exons 7 and 8) genomic sequence that lies between the two inserted *loxP* sites. The *Trpml3^+/−^* progeny were then mated to obtain *Trpml3^−/−^* mice (termed *Mcoln3^tm1.1Jga^* by the Mouse Genomics Informatics).

For Southern-blot analysis genomic DNA was extracted from the selected expanded ES cell clones, then run out on a gel following digestion with *Ssp* I and *BamH* I or *EcoR* I restriction enzymes, and transferred to a nitrocellulose membrane, then hybridized with radiolabeled 5′ or 3′ cDNA probes. The 5′ probe includes exon 3 of *Trpml3* and detects a 5.4-kb band in the wild type allele and a 9.7-kb band in the targeted allele. The 3′ probe includes exons 9 and 10 of *Trpml3* and detected a 10.1-kb band in the wild type allele and a 7.65-kb band in the targeted allele.

To genotype the wild-type [*Trpml3^+^*], floxed [*Trpml3^Flox^*], and knockout [*Trpml3^−^*] alleles we performed PCR amplifications from genomic DNA using 3 genotyping primers: one forward primer [both(F) 5′- GTGGAGCCTTGACTGTCTAG-3′] and 2 reverse primers [wt(R) 5′-CTGTGAGACCTCTTAACAACTCT-3′ and ko(R) 5′-GAACTCTCTCGATCTAACCACTC-3′]. Expected PCR band sizes are 263 bp, 349 bp, and 364 bp, respectively.

### Calculation of relative body weight and growth rate

We weighted mice every day from birth (P0) until weaning (P21) and then weekly until sexual maturity (P64). In order to account for differences in mother's care and litter size, we normalized each animal's weight to the average body weight of the respective litter's wild-type and heterozygote pups. For estimating the relative weight of *Trpml3^−/−^* and *Trpml1^−/−^* mice, we mated respectively *Trpml3^−/+^*×*Trpml3^−/+^* and *Trpml1^−/+^*×*Trpml1^−/+^* mice and compared the resulting *Trpml3^−/−^* and *Trpml1^−/−^* mice with their littermates. For estimating the relative weights of *Trpml3^−/−^; Trpml1^−/−^*, we mated *Trpml3^−/−^;Trpml1^−/+^*×*Trpml3^−/−^; Trpml1^−/+^* and compared the resulting *Trpml3^−/−^;Trpml1^−/−^* with their *Trpml3^−/−^* littermates. Because the relative weights of *Trpml3^−/−^* increases with respect to that of wild type littermates after weaning, we adjusted the relative weights of *Trpml3^−/−^; Trpml1^−/−^* mice after weaning by multiplying their relative weights times the average relative increase in weight of *Trpml3^−/−^* mice at that stage, so that the plotted relative weights (Fig. 9E) of *Trpml3^−/−^;Trpml1^−/−^* can be compared with those of wild type mice. For calculating the daily growth rate (Fig. 9F), we calculated the percent growth increase for each animal with respect to the growth it had the day before, and then averaged the percent growth per genotype.

### Antisera characterization

Our present study uses triple controlled immunohistochemistry to determine the tissue and subcellular expression pattern of TRPML3 protein. We employ antisera raised against distinct regions of TRPML3 We also compare TRPML3 immunoreactivities to available in situ hybridization (ISH) analyses. Finally, we determine which immunoreactivities are absent from the tissues of a *Trpml3^−/−^* mouse. See [15] for detailed TRPML3 antibody information and complete prior characterization. Antibodies used in this study include: TRPML3-NT (rabbit polyclonal, Sigma Cat. # M7570, Lot # 067K4822); LAMP1 (rat IgG2a monoclonal, clone 1D4B, University of Iowa's Developmental Studies Hybridoma Bank, described in [15]) and early endosome antigen 1 or EEA1 (C-15; Goat polyclonal IgG, Cat. # sc-6414, Lot # 0408, Santa Cruz Biotechnology).

### Tissue processing

We used unfixed tissue for in situ hybridization. These tissues were dissected, embedded in OCT and immediately snap frozen in isopentane cooled with dry ice. For fixed adult tissues, we cardiac perfused the animal with 2% paraformaldehyde, dissected out the organs, postfixed for 1 hour in 2% paraformaldehyde, and rinsed 3 times in 1× PBS. We then took the tissue through a sucrose gradient (1 hour each 5%, 10%, 20%) ending with an overnight incubation in 20% sucrose and 50% OCT (Tissue-Tek, Sakura). We mounted the tissue in OCT and froze it on dry ice. We also prepared our own paraformaldehyde fresh from powder. Consistency in fixation was very important as we often saw background autofluorescence that we could ascribe to over or inconsistent fixation.

### Immunohistochemistry

We performed all immunohistochemistry using the ABC/DAB (avidin-biotin complex with diaminobenzidine reaction) signal amplification system (Vector). Following are brief protocols of our immunohistochemistry techniques as previously described in [15].

#### ABC+DAB signal amplification

Sections were postfixed for 10 minutes in freshly prepared 2% paraformaldehyde. Antigen retrieval was done by incubating sections in 10 mM sodium citrate, pH 6 with 0.25% Triton for 20 min at 92°C and cooled for 30 min at room temperature. Quench endogenous peroxidase by incubating in 1% H_2_O_2_,10% methanol. Block for 2 hours in 10% normal goat serum, in 1× PBS. Incubate primary antibody (NT, 1∶2000) in 10% normal goat serum block+0.1% triton (no azide) overnight at 4°C. The following day, rinse 4×10 min in 1× PBS+0.1% triton and incubate with biotinylated secondary antibody (1∶200, goat anti-rabbit, Vector) in 10% normal goat serum block+0.1% triton for 1 hr. Rinse in 1× PBS+0.1% Triton, 50 rpm. Incubate in ABC solution (Vector) for 1 hr, rinse and incubate with DAB solution (Sigma) for at least 7 min until tissue turns light brown. Rinse in 1× PBS. Sections were incubated in DAPI (1 µM) for 10 min to visualize the nuclei under fluorescence. Mount using Prolong Gold (Invitrogen). See [15] for a detailed protocol for the staining.

### In situ hybridization

We performed in situ hybridization on cryostat sections of snap-frozen, unfixed tissues from CD1, *Trpml3* ^+/+^, and *Trpml3* ^−/−^ mice using protocols previously described [16], [59], [60]. Freshly dissected and unfixed tissues were immediately snap frozen by dipping in isopentane cooled to −30°C with dry ice and sectioned (10–12 µm). For ISH, we used two non-overlapping cRNA probes for mouse *Trpml3* mRNA (Genbank ID NM_134160). These are a 5′ probe, which corresponds to nucleotides 179–723 (from codon 60 at the end of exon 1 to codon 240 at the end of exon 5) and a 3′ probe, which corresponds to nucleotides 1005–1594 (from codon 335 in the middle of exon 8 to codon 531 in the middle of exon 12, the last exon).

We PCR amplified these cDNA fragments from mouse inner ear or CVP mRNA and TA-cloned them into vector pCRII. We generated digoxigenin-labeled antisense and sense (control) cRNA probes using the DIG-RNA labeling kit (Roche) according to the manufacturer's instructions. Sections were hybridized with antisense or sense probes as previously described [60]. Sections were mounted for observation. Only cell types that labeled with both the 5′ and 3′ *Trpml3* probes were considered positive for *Trpml3* mRNA.

The *Trpml1* cRNA 3′ *in situ* probe is 463 bp in length corresponding to nucleotides 1179–1641 (from exon 9 to exon 12 of the *Trpml1* mRNA). The *Trpml2* cRNA 5′ *in situ* probe is 506 bp in length corresponding to nucleotides 228–733 (from exon 2 to exon 5 of the *Trpml2* mRNA).

### Image acquisition and analysis

We acquired images using either a Nikon E600 pan fluorescence microscope (20× 0.75 N.A., 60× 1.4 N.A., or 100× 1.4 N.A. objectives) equipped with a CCD camera (SPOT RC-Slider) or a Zeiss LSM 510 confocal microscope (63× 1.4 N.A. or 100× 1.46 N.A. objectives). Or a Leica SP5 confocal microscope (63×, 1.4 N.A. objective) When comparing wild type and knockout immunoreactivities, we captured images under identical conditions. In practice, this meant capturing images with identical exposure settings (pan fluorescence) or identical laser and gain settings (confocal). For even illumination, we flat field corrected and white balanced the color (SPOT RC-Slider) camera prior to acquiring DIC images.

Post acquisition, we identically processed image pairs of wild type tissues and their corresponding knockout controls. This included adjustment for brightness and contrast of all images. We used ImageJ for all post acquisition processing.

### RT-qPCR

RT-qPCR (reverse transcription of RNA followed by quantitative polymerase chain reaction) was performed based on [15]. Specifically, we used tissues from freshly killed animals not cardially perfused. All tissues were dissected as quickly and as cleanly as possible and immediately snap frozen on dry ice until homogenized in Trizol (Invitrogen). We homogenized all tissues in Trizol using a tissue homogenizer and performed RNA isolation according to the manufacturer's instructions. RNA concentration was determined by UV absorption (OD_260_). This value helped determine the volume of RNA used per RT reaction, with the goal to reverse transcribe 1 µg of total RNA per reaction. Prior to reverse transcription, we subjected the 1 µg of total RNA to DNaseI treatment (Invitrogen) to eliminate genomic DNA according to the manufacturer's protocol. This DNaseI treated 1 µg of total RNA was then subjected to first strand cDNA synthesis using Superscript III reverse transcriptase (Invitrogen) according to the manufacturer's protocol.

We performed RT-qPCR using a Mastercycler Realplex2 machine (Eppendorf) on ∼100 ng (2 µl of a 26 µl RT reaction) of first strand cDNA using SYBR Green PCR Mastermix (Applied Biosystems) in triplicate, according to the manufacturer's instructions. The following primers (IDT) were designed on mouse sequence and used in qPCR: Trpml3ex8f, 5′ ATGGAGTTCATCAACGGGTG; Trpml3ex9r, 5′ ATAGTTGACGTCCCGAGAAG; 18Sf, 5′ TTGACGGAAGGGCACCACCAG; 18Sr,5′ GCACCACCACCCACGGAATCG. Melting curve analysis and gel electrophoresis of PCR products indicated single products of the correct size for each primer pair used. In addition, the *Trpml3^−/−^* mouse does not contain the binding site for primer ex8f. Prior qPCR analysis [15] on *Trpml3^−/−^* mice using primers ex8f and ex9r did not detect any product from *Trpml3^−/−^* tissue.

### Tissue histology

Pups were euthanized by decapitation and intestines dissected out and placed in ice cold PBS, separated from their attached connective and vascular tissue, their lumens flushed with PBS, then fixed overnight at 4°C. For frozen sections, tissue was fixed with 4% PFA, washed with PBS, embedded in OCT, snap frozen and sectioned at 8 µm thickness with a cryostat. For paraffin sections, tissue was fixed with 10% neutral buffered formalin, placed in 70% ethanol, dehydrated in increasing series of alcohol, cleared in xylenes or Citrisolv and placed in two subsequent 55°C paraffin baths, embedded and sectioned at 5 µm thickness with a microtome.

#### Hematoxylin and eosin staining

Slides containing paraffin sections were deparaffinized and rehydrated with tap water, stained with hematoxylin solution (Sigma) for 90 seconds, washed continuously with running tap water for 2 minutes, placed in distilled water, dehydrated through an alcohol series, dipped 5 times in Eosin (Sigma) bath, washed immediately in 3 baths of 100% ethanol, cleared with Xylenes and coverslipped.

#### Periodic Acid Schiff staining

Slides containing paraffin sections were deparaffinized and rehydrated to tap water, oxidized in 0.5% periodic acid solution for 5 minutes, rinsed in distilled water, placed in Schiff reagent for 15 minutes, washed in lukewarm tap water for 5 minutes, counterstained in hematoxylin solution for 1 minute, washed in tap water for 5 minutes, dehydrated through an alcohol series, cleared with Xylenes and coverslipped.

### Electron microscopy

A 0.5 cm segment of the distal ileum was dissected in ice-cold PBS, chopped into several smaller pieces and fixed overnight at 4°C with 2.5% glutaraldehyde in 0.1 M sodium cacodylate buffer (pH = 7.2) [for P0 samples] or 2% PFA plus 2.5% glutaraldehyde in 0.1 M sodium cacodylate buffer [for P5 samples]. Tissue samples were stained in osmium solution for 1 hour, embedded in resin, placed in beem capsule and baked overnight in 60°C oven. First, sections were cut at 1 µm, stained with 0.5% toluidine blue on a hot plate for 20–30 seconds, rinsed with dH_2_O and air dried. Then, specimen block was shaved down, ultra-thin sections cut at 70 nm thickness, placed on a grid, stained for 10 minutes with uranyl acetate and lead citrate, allowed to dry, and imaged using the FEI Tecnai Spirit G2 120 kV electron microscope at Northwestern's Nikon Cell Imaging Facility.

### Oil Red O staining

Dissected 1 cm segmentS of proximal and distal small intestines were fixed overnight with 4% paraformaldehyde, washed in 1× PBS, dried off excess moisture and snap frozen in OCT using chilled isopentane dry ice bath. Oil Red O was dissolved at 0.5% in propylene glycol while gently heating to 95–100°C, filtered through 25 µm filter paper while still warm and allowed to cool. Slides containing frozen sections were air and vacuum dried, post-fixed 5 minutes, then air dried, placed in absolute propylene glycol for 3 minutes, stained 10 minutes in pre-warmed Oil Red O solution in a 60°C oven, washed in 85% propylene glycol solution for 5 minutes, washed in distilled water and counterstained with hematoxylin for 60 seconds, washed in running tap water and coverslipped with aqueous mounting media.

### Dextran uptake

P0 pups were removed from the mother right after birth -prior to receiving any feeding from the mother- while older pups (P6) were removed from the mother and held in a warm chamber for 1 hour. The animals were then fed, 10 ul per 1 g weight, with commercially available infant formula (Enfamil, 1 scoop per 2 fl oz water, warmed to 37°C.) with Texas Red-conjugated dextran (Life Technologies) at 1 mg/ml. For P6 pups, the animals were returned to the mother overnight before dissection at P7. For P0 pups, the animals remained isolated from the mother and kept in 30°C environment for 3 hours before dissection. After the dissection, intestinal tissues were fixed overnight in 4% paraformaldehyde, washed in 1× PBS, dried off excess moisture and snap frozen in OCT using a dry ice-chilled isopentane bath.

We performed immunohistochemistry using the ABC/DAB signal amplification system (Vector) on frozen sections from Dextran-fed animals using the protocol described above with slight modifications for LAMP1 and EEA1 staining. Specifically, we omitted the antigen retrieval step. For LAMP1, sections were incubated in primary antibody at 1∶50 and biotinylated secondary antibody at 1∶500 (goat anti-rat, Santa Cruz Biotechnology). For this specific staining, DAB with metal enhancer (Sigma) was used, giving dark grey signals rather than brown signals from traditional DAB. For EEA1 staining, 10% donkey normal serum was used. Sections were incubated in primary antibody at 1∶100, and biotinylated secondary antibody at 1∶200 (rabbit anti-goat, Vector).

In addition, we also performed immunofluorescent staining on these sections for LAMP1 and EEA1 based on the staining described above with modifications. Specifically, we incubated the sections in 1 mM glycine for 30 minutes and rinsed 3 times in 1× PBS after post fixation in an attempt to quench some of the autofluorescence caused by aldehyde fixative. We also added a permeabilization step in which the tissues were incubated in 0.1% tritonX in 1× PBS for 30 minutes. For LAMP1, sections were then incubated in primary antibody at 1∶50 and secondary antibody at 1∶500 (DyLight 405 Goat anti-rat, Jackson ImmunoResearch) and goat serum was used for blocking step. For EEA1 staining, sections were incubated in primary antibody at 1∶100, and secondary antibody at 1∶200 (DyLight 405 Donkey anti-goat, Jackson ImmunoResearch). The 405 nm signals were pseudocolored green for clearer visualization.

The amount dextran endocytosis was determined by the total intensity of Texas Red fluorescence per cell. To achieve this, z-stack series of images were taken using from frozen sections mounted in Prolong Gold (Invitrogen) without post fixation using Leica SP5 confocal microscope (63×, 1.4 N.A. objective). All confocal laser intensities and exposures remained identical for all samples. The images were taken every 0.5 um for the entire thickness of the frozen section. The measurement and analysis for the amount of dextran uptake in the enterocytes were done on imageJ using protocols adapted from [61], [62]. In brief, Texas Red fluorescence density was individually measured and combined from each optical section spanning the thickness of a single cell. To calculate total fluorescence per cell per optical section, we followed this equation: Corrected total fluorescence per cell = sum of florescence density per cell−(area of selected cell×mean fluorescence of background readings).

### Transcytosis

We biotinylated mouse IgGs (Jackson Immuno Cat # 015-000-002) and, for control, chicken IgYs (Jackson Immuno Cat # 003-000-002) from gamma globulin samples that were 98% IgG or IgY using the EZ Link kit (Pierce). We removed the free biotin from the biotinylation reaction mixture by column-purification following the manufacture's protocol, and assayed for biotin incorporation by reducing the IgG and IgY, running on an SDS-PAGE and Western blotting with Avidin-HRP (1∶5000), and by ELISA as described below for the serum samples. Biotinylated IgG concentration was 8 µg/µl.

Pups were separated from the mother, kept on a warming pad and fasted for 1 hr prior to mouth feeding with a pipette. Biotinylated mouse IgG (or chicken IgY as negative controls) were mixed in equal volumes with formula (Enfamil, 1 scoop per 2 fl oz water, warmed to 37C) to a final concentration of 4 ug/µl, and fed to pups at 10 µl per gram of body weight. Fed pups were returned to their mothers for 6 hours prior to blood collection from the heart (using a 26 G needle) under Ketamine+Xylazine anesthesia, and serum prepared and stored frozen. Biotinylated IgG and IgY levels were estimated by ELISA as follows: serum was immobilized on plates coated with goat anti-mouse or (for negative controls) goat anti-chicken antibodies (Jackson Immuno), probed with avidin-HRP (100 µl per well at 0.1 µg/ml), developed with ABTS solution (100 ul/well, Sigma) and images with an ELISA reader.

## Supporting Information

S1 Figure Restricted expression of *Trpml3* mRNA in cells of neonatal intestine, kidney, skin, lung thymus, olfactory epithelium and vomeronasal organ. *In situ* hybridization with a probe to *Trpml3* (complementary to 3′ portions of its mRNA) to sagittal sections of P2 mice reveals strong mRNA levels in: (A) thymus (Th); (B) chemosensory neurons of vomeronasal organ (VNO) and olfactory epithelium (OE) as well as presumed skin melanocytes close to hair shafts (H); (C) scattered cells of lung (Lu) presumed to be alveolar macrophages (bronchioles are labelled as B); and (D) epithelia of intestinal villi and cells of kidney presumed to be principal cells of the collecting duct. We also detected *Trpml3* mRNA in epithelial cells of inner ear [15], but nowhere else in these neonatal whole-mouse sections.
(TIF)

S2 Figure Representative morphological comparison between wild type, *Trpml1^−/−^, Trpml3^−/−^* and *Trpml3^−/−^;Trpml1^−/−^* postnatal P7 enterocytes. Tissues were collected from P7 pups fed with Texas Red-dextran. (A,D,G,J) H&E staining for gross morphological examination of P7 intestinal villi. (B–C,E–F,H–I,K–L) Electron micrographs for ultrastructural examination of intestinal enterocytes. These micrographs indicate that there are both lysosomes (L) and giant lysosomes (GL) with similar distribution and morphology between wild type (A–C),*Trpml1^−/−^* (D–F) and *Trpml3^−/−^* (G–I). The lysosomes and giant lysososomes contain large amount electron-dense materials, presumably dextran (C,F,I). The large pathological vacuoles (V) are only present in *Trpml3^−/−^;Trpml1^−/−^* (J–L) and, unlike the giant lysosomes in the controls, appear mostly electronluscent and take up most of the cytosolic space. Scale bars indicate 50 µm for H&E images and 2 µm for electron micrographs. Organelles present in control enterocytes are labeled in blue, whereas pathological structures are in pink. (N, nucleus).
(TIF)

S3 Figure The pathological vacuoles of suckling enterocytes lacking mucolipins 1 and 3 are not filled with fats, glycosaminoglycans and polysaccharides. (A,B,E,F) Oil Red O staining of frozen sections from P7 intestines reveal lipid deposition in the lacteals of both (E,F) control and (A,B) *Trpml3^−/−^;Trpml1^−/−^* littermates, but not in their pathological vacuoles. (C,G) Periodic Acid Schiff (PAS) staining of sections from P7 intestines reveal glycosaminoglycan accumulation in the goblet cells of both (G) control and (C) *Trpml3^−/−^;Trpml1^−/−^*littermates, but not in their pathological vacuoles. (D,H) Toluidine Blue staining of sections from P0 intestines reveal polysaccharide accumulation in the goblet cells of both (H) control and (D) *Trpml3^−/−^;Trpml1^−/−^* littermates, but not in their pathological vacuoles.
(TIF)

S4 Figure Mucolipin co-deficiency does not alter the rates of endocytosis or transcytosis in neonatal enterocytes. (A,B) Confocal projection image of enterocytes from P0 littermates fed Texas Red-conjugated dextran right after birth, 120 minutes prior to fixation. (A) Two enterocytes from control *Trpml3^−/−^* pups. (B) Two and a half enterocytes from *Trpml3^−/−^;Trpml1^−/−^* pups. (C) Average cellular levels of endocytosed dextran, calculated from 3 pups per genotype. For each animal, the value used is an average from 10 to 20 enterocytes. Error bars indicate SEM. P value was calculated with a Student's t-test. Despite the different subcellular distribution of dextran, cells from both genotypes have endocytosed similar amounts of it. (D) Average apical-membrane endocytic figures observed in electron micrographs of enterocytes of control *Trpml3^−/−^* and *Trpml3^−/−^;Trpml1^−/−^*littermate pups at P0. Sample size was 3 per genotype. Error bars indicate SD. P value was calculated with a Student's t-test. Despite the incipient vacuolation of *Trpml3^−/−^;Trpml1^−/−^* enterocytes (see figs. 4 to 6), their rate of apical endocytosis plus exocytosis, as assessed from their number of endocytic figures, do not differ from those of *Trpml3^−/−^* controls. (E) Transcytosis of maternally fed antibodies does not differ between *Trpml3^−/−^;Trpml1^−/−^* pups and control *Trpml3^−/−^* littermates (P8-11). Animals were fed formula alone (negative control #1) or formula containing biotinylated mouse IgG (which is internalized by FC-receptor mediated endocytosis at the apical membrane of suckling enterocytes and exocytosed basolaterally into the lymphatic circulation). As a second negative control, some pups were fed biotinylated chicken IgG, which is not recognized by the mouse FC receptor. Six hours after feeding, serum levels of biotinylated antibodies were measured by ELISA after immobilization on plates containing goat anti-mouse (or, for the second negative control, goat-anti-chicken) and visualized via avidin-HRP. As expected, biotinylated antibodies were detected in wild type mice fed mouse IgG but not on mice fed chicken IgY or formula alone. However, the amount of transcytosed biotin-IgG did not differ between genotypes: Student's t-test P = 0.8 between *Trpml3^−/−^;Trpml1^−/−^* (n-3) and wild type (n = 5), and P = 0.4 between *Trpml3^−/−^;Trpml1^−/−^* (n-3) and *Trpml3^−/−^* (n = 3). Error bars indicate SD.
(TIF)
